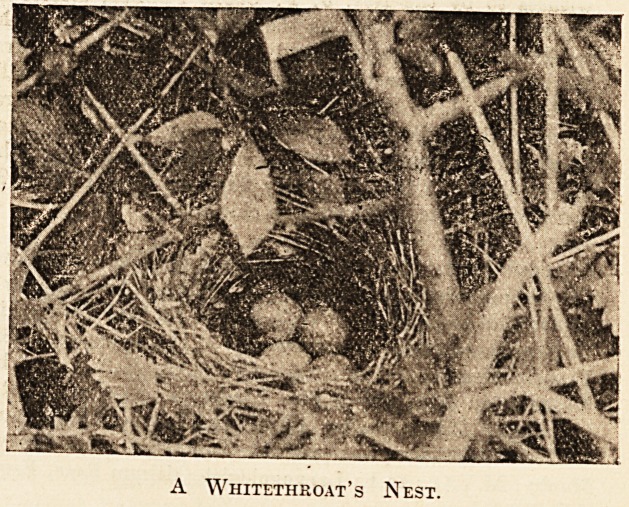# A Bird-Watcher's Camp—III

**Published:** 1910-10-01

**Authors:** 


### The Practitioner's Relaxations.

### A BIRD-WATCHER'S CAMP—III.

The skill displayed by birds in building their nests—weaving and plaiting with no better tools than their bills—is one of the miracles of Nature. They seem never to make serious mistakes and they work at an extraordinary speed. I had the good fortune during my camping time to watch the development of a whitethroat's nest from its first beginnings. By a stroke of luck I saw the bird slip into the tangled herbage at the foot of a hedge, carrying a dry stalk of grass in its bill. I marked the spot, waited till the bird flew out again, then very gently put the herbage aside till I found about two dozen stalks laid one across the other about nine inches from the ground. This was on June 13. I visited the spot again on June 15 and found the deep cup-like nest outwardly complete and partially lined. On the 16th the lining seemed to be complete. On the 17th I could see no change. On June 18 there was one egg in the nest, and on June 21 there were three, and the bird was sitting—as often happens in the case of second broods, the clutch was a small one. On July 11 there were two young birds in the nest, the third egg having disappeared. I had not visited the nest for some days, and was therefore unable to say how long the young had been hatched, but they appeared to be some four or five days old. If I am right little more than three weeks elapsed between the laying of the first foundations of the nest and the hatching of the young.

My friend the gamekeeper gave me a lot of useful information, but I was sometimes inclined to suspect him of drawing the long-bow. Evidently I failed to conceal my suspicions altogether, as the story I am about to tell will show. Naturally our conversation turned frequently on the subject of partridges, and one evening he asked me whether I had ever seen a pair of partridges sitting together on the nest. I had not, and said so. He then told me that, when the young were hatching, the cock bird generally joined the hen on the nest. I did not think this was altogether improbable, but he evidently detected some traces of a lingering scepticism in my replies. He had lately helped me to photograph a sitting partridge. A few days later, on my return to camp in the evening his wife met me with a request that I would take my camera at once to that nest. "The young birds are hatching," she said, "and both birds have been on the nest all day. Steward (the keeper's name) particularly wished you to see them and photograph them if you could, because he said he knew you did not believe what he told you about it." I obeyed at once and found the two birds sitting side by side with their heads pointing in opposite directions. I even secured a photograph of them, but I was afraid to do much in the way of exposing them to view and the photograph does not, therefore, illustrate the phenomenon distinctly. It is an interesting record nevertheless.

The rough notes I kept in camp show that I had about fifty different species of birds more or less under observation while I was there. None of them were particularly rare, but there is much to be learnt about the commonest birds and I became fairly intimate with a good many of them. To secure any degree of intimacy with birds one must cultivate the faculty of standing, sitting, or lying motionless and of very slow movement when occasion requires. I have had normally shy birds feeding unconcernedly within a few feet of me, and by turning my head very slowly have been able to follow them with my eyes as they moved from place to place. Field glasses are, of course, invaluable to a bird-watcher, but it is infinitely more satisfactory, where possible, to watch the birds at close range with the naked eye. As I wandered silently about the neighbourhood of my camp I made many interesting and some curious observations, but, as I have already said, I made no great discovery. Of the curious things I saw, perhaps the most comical was a common wren sitting in a disused nest, probably that of a linnet. It was in the evening. The nest was in a somewhat exposed position, and I saw the bird sitting before she saw me. Until she flew off the nest I was quite at a loss as to what the bird was. When she proved to be a wren, and was, moreover, joined by a second wren, I thought I had come across a marvellous novelty—a wren incubating on an open nest. But the nest proved to be empty, and I passed on mildly amused at the bird's eccentric choice of what evidently was no more than a roosting-place. When I had walked a few hundred yards further curiosity tempted me to return to the spot, and there I found the two birds again, one sitting on the nest as before and the other perched close by. The sitting bird again vacated the nest, betraying all the symptoms of irritation common to wrens. I visited the nest on several occasions subsequently, but I never found it tenanted again. Probably the wren was a young one and had selected the nest merely as a convenient place to pass the night in.

I am afraid I was most happy when I was most idle. I used to lie for hours just inside or just outside my tent listening to or watching the birds about it. There was an oak tree of which I could command a view as I lay in bed which I named "Tree-pipit Oak" because it was the favourite perching-place of a tree-pipit who was one of the best performers I ever met of all his sweet-voiced family. His nest was undoubtedly close by in the grass; but I did not find it—indeed, I hardly looked for it. It was enough for me to watch his soaring flights and enjoy his varied song, and especially the long soft musical notes that accompanied his downward flight as he swung, fluttering like a falling leaf, back to his perch.

A spotted fly-catcher also had a nest close by and a watch-tower in the form of a dead stump in front of my tent. From dawn till dark he was busy with his beneficently destructive labours—I once watched him at work as late as 9 p.m.—and, on a still day, I could often hear the snap of his bill as he seized a passing fly or moth. And if my eyes became tired with watching I could close them and still enjoy the sounds of life about me, the crooning of the turtle-dove, the twitter of the linnets and greenfinches in

A Whitethroat's Nest.

the bushes, the half-merry, half-plaintive cadence of the willow-wrens, the evening carols of thrushes and blackbirds. These and innumerable other sounds I marked evening after evening, noting how, one by one, they died away as night drew down her veil, till the hoot of a distant owl and the soft breeze coming fresh and cool over the dewy grass alone broke the stillness of the night.

## Figures and Tables

**Figure f1:**